# Kalman Filter-Based Fusion of LiDAR and Camera Data in Bird’s Eye View for Multi-Object Tracking in Autonomous Vehicles

**DOI:** 10.3390/s24237718

**Published:** 2024-12-03

**Authors:** Loay Alfeqy, Hossam E. Hassan Abdelmunim, Shady A. Maged, Diaa Emad

**Affiliations:** Mechatronics Engineering Department, Faculty of Engineering, Ain Shams University, Cairo 11535, Egypt; hossameldin.hassan@eng.asu.edu.eg (H.E.H.A.); shady.maged@eng.asu.edu.eg (S.A.M.); diaa.emad@eng.asu.edu.eg (D.E.)

**Keywords:** sensor fusion, bird’s-eye-view, multi-object tracking, structure similarity, data association, Kalman filter, self-driving cars

## Abstract

Accurate multi-object tracking (MOT) is essential for autonomous vehicles, enabling them to perceive and interact with dynamic environments effectively. Single-modality 3D MOT algorithms often face limitations due to sensor constraints, resulting in unreliable tracking. Recent multi-modal approaches have improved performance but rely heavily on complex, deep-learning-based fusion techniques. In this work, we present CLF-BEVSORT, a camera-LiDAR fusion model operating in the bird’s eye view (BEV) space using the SORT tracking framework. The proposed method introduces a novel association strategy that incorporates structural similarity into the cost function, enabling effective data fusion between 2D camera detections and 3D LiDAR detections for robust track recovery during short occlusions by leveraging LiDAR depth. Evaluated on the KITTI dataset, CLF-BEVSORT achieves state-of-the-art performance with a HOTA score of 77.26% for the Car class, surpassing StrongFusionMOT and DeepFusionMOT by 2.13%, with high precision (85.13%) and recall (80.45%). For the Pedestrian class, it achieves a HOTA score of 46.03%, outperforming Be-Track and StrongFusionMOT by (6.16%). Additionally, CLF-BEVSORT reduces identity switches (IDSW) by over 45% for cars compared to baselines AB3DMOT and BEVSORT, demonstrating robust, consistent tracking and setting a new benchmark for 3DMOT in autonomous driving.

## 1. Introduction

The realization of autonomous vehicles hinges on their ability to accurately perceive and track objects in their surroundings. From parking to driving on the highway and maneuvering in high-traffic conditions, autonomous vehicles need to actively respond and interact with surroundings in a real-world driving scenario. The key capability that enables an autonomous vehicle to reach such a level of automation and accuracy is the perception of the surroundings, its ability to detect and track objects to be able to plan a safe maneuver to the goal destination. Detecting objects is the task of recognizing surrounding objects, identifying their category and determining their location and size, while tracking objects is about associating detected objects between consecutive frames and assigning a unique ID for each object to use later for re-identification. Object tracking can also estimate motion trajectory by modeling the dynamics of the tracked object to predict its next motion.

Accurate 3D multi-object tracking is a critical component of the perception stack for autonomous vehicles. Typical use cases include collision avoidance situations, where a vehicle can track surrounding objects by predicting their trajectory, then estimating Time to Collision (TTC) with each potential target in the driving area. Collision avoidance is one example of several features developed in Advanced Driving Assistance Systems (ADAS) that aim to provide a safe and seamless driving experience. These kinds of features typically do not rely on one sensor to track the object, but multiple sensors can cooperate to provide such information. The signals from these sensors are distributed by noise, and the modeling of this noise should take the operating conditions into account.

Multi-object tracking (MOT) is a more challenging task compared to single object tracking. MOT requires simultaneous updates for each object, which increases the computational complexity, considering both time and memory. In addition to computational complexity, MOT is prone to confusing or misdetecting objects. Highlights of challenges for MOT that make it a hard problem to solve include the following:

- Object similarity: Distinguishing objects that share a common shape or have a similar appearance is a difficult task for MOT, especially when considering pedestrians with similar clothes, cars with the same color and nearly the same shapes, which leads to a common problem known as ID switching. In such cases, MOT algorithms need to be able to perceive visual cues or deduce motion patterns to overcome this challenge.
- Occlusion: Objects closer to the observing sensor can block other objects that are far away from the sensor, which can become partially or fully invisible in the sensor field of view, depending on the overlapping size. This can happen in high traffic scenarios. In such cases, MOT algorithms need to be robust enough to overcome such limitations.
- Sensor limitations: Sensors can be modeled with some assumptions to relax hard constraints that are not feasible to be modeled or cannot be simply approximated, which can lead to model uncertainties. Also, each sensor has limitations perceiving the environment under some conditions. Typical cameras struggle in low-light conditions or due to high exposure to light, while LiDAR can have difficulty with high reflective surfaces or during rains. MOT algorithms need to consider limitations of the sensors and leverage the power of sensor fusion to overcome these challenges.
- Clutter: Measurements that interfere with the target object and get misinterpreted can cause it to be seen as clutter. This occurs in cases where nearby objects trigger measurements that the sensor can detect as the object of interest but in fact do not belong to the target object. This can increase the false positive (FP) tracks, considering the case of reflections of objects in the mirror or on the car’s windshield that might be detected as objects, or increase the false negative (FN) tracks, where dense measurements can make it hard to detect sparse ones that are close to it.
- Dynamic environment: A dynamic environment adds more difficulty to the task. Some objects such as pedestrians can unpredictably move, making them difficult to model. Changing weather conditions can also affect visibility.

Recent years have witnessed numerous contributions to the 3DMOT challenges. A wide range of techniques have been proposed. These techniques can be divided into categories based on input modality or learning methods. From the modality point of view, there are camera [1,2,3,4,5], radar [6,7,8] and LiDAR [9,10,11,12,13,14]—based methods. The single modality [1,2,3,4,5,6,7,8,9,10,11,12,13,14] category is dependent on the detection accuracy of the sensor, where a biased detector can introduce high false positive (FP) and false negative (FN) tracks, which impacts the tracking performance. Moreover, sensors can fail in certain conditions that make the tracking output unreliable. Sensor fusion-based methods for multi-modality [15,16,17,18,19] leverage information from different sensors and fuse these together, filtering noisy signals and overcoming the individual shortcomings of each sensor modality, resulting in more robust and accurate tracking performance and leading to more reliable and safe autonomous driving features. From a learning-method perspective, a group falls under classical methods [9,10,11,12,13,14] that rely on probabilistic estimation and data association approaches, using a motion model to predict object dynamics and association criteria to assign new measurements to tracks. Often called Tracking by Detection (TBD), this uses a separate model to detect objects and a tracker to associate this detection with tracks, which simply can be abstracted as an association problem.

Sensor fusion-based tracking methods achieve better results, reflecting the different learning methods in this category. We can conclude that, in general, deep learning methods such as [19] achieve better accuracy and generalize better. However, they are computationally expensive, requiring high-end GPUs and a large memory footprint, making them not the ideal option for micro-embedded or on-device inference. The latest classical methods [15,16,17,18,19] show that they can achieve the close performance of deep learning fusion-based methods while using smaller and more efficient classical algorithms. By rethinking and exploiting the design of these algorithms, they can achieve a good balance between accuracy and real-time performance. Moreover, they have predictable behavior, and with good testing, they can be interpreted easily, unlike deep learning approaches that can raise safety concerns, as they are black-box solutions that have the potential for catastrophic failures in unforeseen scenarios. And, finally, they are vulnerable to adversarial samples.

This work exploits classical fusion algorithms, building on top of successful approaches for camera–LiDAR fusion algorithms, using the TBD approach for feeding inputs from camera and LiDAR detection to a bird’s eye view tracker that maintains the 3D state of objects while updating their fused information in BEV space. Key contributions are listed as follows:

- Introducing more robust constraints on data association using Sum Absolute Difference Grid Weighted Average Census (SAD-GWAC) on 2D boxes by dividing each box into a grid, encoding every grid by the weighted average depth value of the corresponding depth value, then matching based on the minimum absolute difference combined with the census Hamming distance of the boxes;
- Using BEVSORT as a tracking framework that modifies the typical Kalman filter state vector to efficiently represent the boxes’ geometry, filter noisy areas and relax the assumption of linear motion dynamics by introducing acceleration as correlated error with the states to account for instantaneous non-linear motion;
- Replacing Intersection over Union (IoU) with Oriented Generalized (OGIoU) as the criterion for association between tracks and measurement;
- Improving existing track management methods using modality fusion with state buffers for unconfirmed and lost tracks to account for IDSW, FPs and FNs.

## 2. Related Work

This work [1] introduces a computationally efficient approach that relies on a Kalman filter with a linear motion model constant velocity (CV) to predict motion in 2D images and simple data association Intersection over Union (IoU) between tracks and measurements by solving a linear sum assignment using the Hungarian algorithm [20]. It fails to reliably track objects under rapid motion, varying appearance or dynamic scenes with high occlusion due to these linear assumptions. Object re-identification remains a challenge that would require additional overhead. Moreover, velocity estimation in pixel space is not accurate compared to direct estimation from Time of Flight (ToF) sensors. This study [5] builds upon SORT framework by extending simple data association to include an appearance similarity metric to handle varying appearances in dynamic scenes, which allows the re-identification of missing tracks by using a neural network for modeling the appearance of objects. It still uses a typical Kalman filter with a CV motion model, which may not be sufficient for rapid motion. It can be sensitive to the quality of appearance features that can be affected by noise, deformation or illumination changes, which can degrade the quality of association. It also estimates velocity in pixel space. Most classical methods apply Non-Max Suppression (NMS) and other confidence threshold filtering approaches to associate non-noisy detection. This study [4] associates every detection box with existing tracks by leveraging boxes with low confidence instead of complete discard. It prioritizes high confidence detection over lower ones by allowing only high confidence detection to merge with the corresponding match with existing tracks to update their state, and tracks that receive no update are considered occluded and candidates for termination by those stored in different buffers meant for tentative tracks. Lower confidence tracks are used to recover these tracks, reducing the re-initialization of occluded tracks. This simple approach is easy to implement for improving tracking accuracy; however, it can be sensitive to false positive detection.

In [9], the authors extended the basic SORT algorithm to accommodate 3D object detections derived from point cloud-based detection models. This adaptation enables the tracking of objects in three-dimensional space, offering a significant improvement over traditional 2D-based tracking methods. It achieves high tracking accuracy and real-time performance due to the simplicity of the model. It is easy to implement and debug, and by using point-cloud detectors, which have more accuracy for moderate-sized objects like cars, it improves the tracking accuracy. However, it can fail with linear motion assumptions, especially for dynamic objects with rapid motion, and has another limitation regarding point-cloud detection models. In general, they are not accurate, with smaller objects increasing false negatives (FN), which in return affect tracking accuracy.

Camera-to-LiDAR projection throws away the semantic density of the camera features, especially with sparse LiDARs. Only a few camera features will get matched to a LiDAR point, so [19] proposed a multi-task, multi-modal, deep learning fusion approach, by extracting each sensor features separately, then fusing them in a unified bird’s eye view (BEV) feature space, which preserves both semantic and geometric information. Such an approach typically suffers from high latency; however, they managed to reduce this view-transformation bottleneck by 40 times, compared to previous methods. By stacking a few task-specific heads to the encoder responsible for fusion, they can enable 3D multi-task learning by the same model. It still carries heavy computational loads involving multiple feature extraction and fusion, making it more challenging to achieve real-time performance on embedded devices, in addition to the high memory footprint.

Most traditional MOT algorithms directly perform data association between detections and tracks to match correspondences, which may not be accurate, as the prediction quality is dependent on the prediction model. In many cases, this brute-force approach underperforms the object dynamics, especially when objects are occluded or undergo a nonlinear maneuver. The study [10] constructed a prediction confidence for each predicted state to adjust the influence of prediction confidence on data association. This adaptively adjusts the search range of data association when prediction confidence decreases. To further improve the data-association method, the proposed pairwise association cost takes into consideration geometry, appearance and motion cost. Geometry cost, unlike ‘IoU’ methods, uses a weighted sum of the boxes’ dimensions, Euclidean distance between centers and orientation shift. Appearance cost is the L2 distance between appearance features extracted from the detector backbone. Existing camera and LiDAR fusion methods are designed with complex feature extractors that are computationally inefficient. On the other hand, ref. [16] established a relatively faster fusion method, aiming for real-time performance. It primarily focuses on 3D tracking of objects, while using 2D tracks as a complementary source of information, with a hierarchical association process. It uses a LiDAR 3D object detector model to acquire 3D detections and a 2D camera object detector to obtain 2D detections. Then, by projecting 3D boxes into the image, it can convert them to 2D boxes and obtain an IoU score between the projected 2D LiDAR detections and the 2D camera detections in image space to perform fusion between both detections. The major contribution is in their four levels of association method. For overlapping detections, [15] projected them onto a BEV-plane, using a technique suggested in [21] to estimate the depth of the 2D box center from neighbor points in a depth map patch corresponding to the box location in the image, which makes this approach able to split the occluding detections in the image plane.

The selection of the linear Kalman filter (KF) in the CLF-BEVSORT framework reflects a deliberate balance between computational efficiency and tracking performance, tailored to the real-time requirements of autonomous vehicle systems. The linear KF, based on a constant velocity (CV) motion model, is particularly well-suited for the bird’s eye view (BEV) tracking space, where object motion is predominantly linear or near linear. This simplicity ensures that the computational demands remain manageable, making it ideal for embedded systems with limited processing power. Moreover, linear KF achieves these results without sacrificing the accuracy required for high-performance multi-object tracking (MOT), as evidenced by its state-of-the-art results on the KITTI dataset.

In contrast, advanced tracking methods such as the Particle Filter (PF), Extended Kalman Filter (EKF) and Unscented Kalman Filter (UKF) offer more sophisticated handling of non-linear dynamics, but at a cost. PF, for instance, excels in managing non-Gaussian noise and multi-modal distributions, making it highly versatile for complex tracking scenarios. However, its reliance on processing a large number of particles significantly increases resource demands, making it impractical for real-time applications in resource-constrained environments like CLF-BEVSORT. Similarly, UKF provides superior accuracy for non-linear systems by propagating sigma points to approximate state distributions, avoiding the linearization errors inherent in the EKF. Despite this, UKF introduces substantial computational overhead, requiring extensive matrix operations to handle the propagation of sigma points and update equations. These complexities limit the UKF’s suitability for real-time embedded applications, where low latency is critical.

The Interactive Multiple Model (IMM) framework combined with the Unscented Kalman Filter (UKF) is a robust approach for handling multi-object tracking in scenarios with diverse motion dynamics. IMM dynamically switches between multiple motion models, such as constant velocity (CV), constant acceleration (CA) or constant turn rate and velocity (CTRV), based on the observed behavior of tracked objects. The use of the UKF enhances the IMM framework’s ability to handle non-linear dynamics, as it avoids the linearization errors inherent in the Extended Kalman Filter (EKF). This combination makes IMM-UKF particularly suitable for tracking objects with abrupt motion changes or highly non-linear trajectories. Despite its robustness, the IMM-UKF approach introduces additional computational overhead due to the simultaneous propagation of multiple models and sigma points, which can limit its applicability in real-time embedded systems. Frameworks like IMM-UKF-PF, which incorporate Particle Filter methods, further extend its utility for multi-modal distributions but increase computational demands even more, making such approaches better suited for off-line evaluations on benchmarks like KITTI rather than real-time applications.

The linear KF, by comparison, effectively captures the motion patterns typical of objects such as vehicles and pedestrians in the BEV space. While higher-order motion models, such as constant acceleration (CA) or constant turn rate and velocity (CTRV), paired with EKF or UKF, could potentially offer improved performance in scenarios with highly non-linear motion, they introduce additional complexity that may not justify the incremental gains for this application. CLF-BEVSORT demonstrates that a simpler motion model combined with the linear KF can achieve robust and accurate tracking without incurring the additional latency and resource consumption of these more advanced methods. Another advantage of the linear KF is its ability to avoid the challenges associated with the approximation errors of the EKF and the computational demands of the PF and UKF, while still providing sufficient accuracy for most real-world tracking scenarios. The relatively straightforward implementation of the linear KF ensures seamless integration within the modular CLF-BEVSORT framework, which prioritizes scalability and efficiency alongside tracking performance. Its ability to achieve precise results with minimal computational overhead underscores its value as a practical and reliable choice for autonomous vehicle applications.

## 3. Methodology

This section outlines the proposed contributions, beginning with a detailed problem formulation that defines the inputs, outputs and key parameters employed in our approach. Following this, we delve into the tracking framework, elucidating the architecture of the models and the underlying mathematical details of the algorithm. Subsequently, we explore the fusion process for combining tracked states from camera and LiDAR inputs. Finally, we discuss the track management process, encompassing state buffer updates and tracker categorization.

### 3.1. Problem Formulation

**Figure 1:**
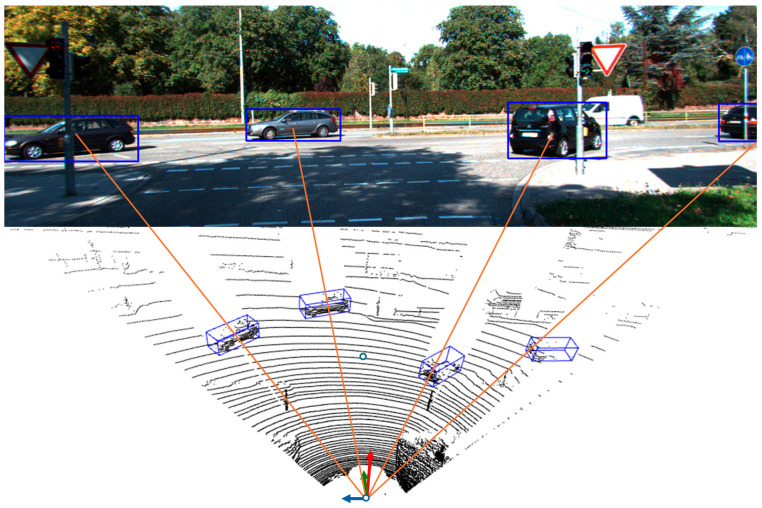
Inputs to our fusion tracking approach, where 3D detections from the LiDAR point-cloud are shown in the lower half of the image, and the camera 2D detections are shown in the upper half. The red lines relate the camera detections to the lidar detections following the camera perspective.

Object tracking is the task of recognizing an object across multiple frames, where an object can be represented as a bounding box in corners format {*x*_1_, *y*_1_, *y*_2_, *x*_2_, *w*, *h*, *s*, *c*} for the 2D case, as shown in Figure 1, where (*x*_1_, *y*_1_) represent the top-left box corner, (*x*_2_, *y*_2_) represent the bottom-right box corner and (*s*, *c*) represent the box score and label, respectively. This can represent 2D camera detected boxes, so *B_c_* is the set of N boxes: *B_c_ =* {*B_c_^i^|*1 *≤ i ≤ N*} given a camera input as *I*(*W*, *H*, 3), where *W* is the image width and *H* is the image height. For the 3D case, the object is represented with a 3D bounding box, detected by a LiDAR detection model in our case, such that *B_l_* {*x*, *y*, *z*, *l*, *w*, *h*, *θ*, *s*, *c*}; similarly, (*x*, *y*, *z*) represent the box center point in 3D, and (*l*, *w*, *h*, *θ*) represent the 3D box size (length, width, height) and z-orientation (yaw).

Our tracking method uses a BEV map to track objects in bird’s-eye-view perspective by projecting 3D boxes into BEV space, such that a tracked state would be *T*: {*x*, *y*, *r*, *a*, *v_x_*, *v_y_*, *θ*, *s*, *c*, $\dot{a}$, *i*}, where (*x*, *y*) is the bird’s eye view box center location in LiDAR coordinates, (*r*, *a*) represent the box aspect ratio of length to width and the 2D box area, respectively, (*v_x_*, *v_y_*) represent the *x*, *y* velocities and ($\dot{a}$, *i*) are the change in box area and the box’s unique identity number. A Kalman filter is used to update the tracked states in a prediction update scheme. A prediction motion model is used to predict states when data is not available, then associate incoming measurements with a suitable criterion complying with the sensor type.

### 3.2. Model Architecture

The proposed work utilizes both modalities to include accurate 3D position information from the LiDAR with 3D object detector as the main seed for initializing tracks, as LiDAR 3D detectors outperform camera-based methods. On the other hand, semantic information is extracted from the camera to improve the tracking precision while maintaining strong recall of tracks. First, LiDAR detections are projected to the BEV map. Second, a Kalman filter is used as suggested by [14] as the state estimator, with a constant velocity motion model to predict motion when measurement is not available, following the standard SORT framework. It also accounts for the change of velocity as a white noise that perturbs the states, which better merges with measurements that undergo some instantaneous rapid maneuver.

**Figure 2:**
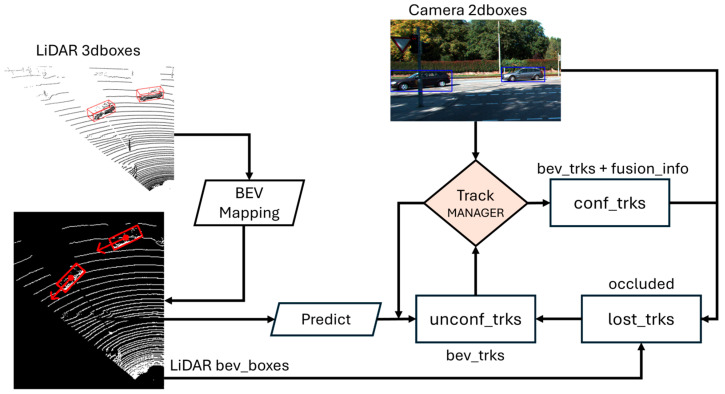
CLF-BEVSORT architecture. Given the camera 2D boxes in blue and the LiDAR 3D boxes in red as inputs, LiDAR 3D detections are projected to the bird’s eye view to initialize/update the tracks with the BEV-states. All valid tracks perform prediction steps using the motion model to estimate new states. Tracks are then projected to the camera and associated with newly detected 2dboxes to confirm track existence, influencing confirmed/unconfirmed/lost tracks handled by the track manager. The tracker controls the birth of new tracks, filtering clutter and updating existing tracks.

The track manager is the core that serves the input, updates the states and is responsible for generating the output, as shown in Figure 2. First, the track manager predicts new states for all existing tracks. Then, it associates the new BEV boxes with existing tracks to update their states (position, orientation, size) only from LiDAR, then performs a second step association with the camera, which updates semantic information (existence score, label). Unmatched BEV boxes with the camera are then considered as unconfirmed tracks waiting for the camera to associate, or a certain threshold of ‘K’ merges with the BEV boxes to move it to confirmed tracks. Tracks that remain unassociated with BEV boxes for M consecutive cycles and have not been fused with camera data are transferred to a lost buffer. This mechanism helps minimize identity switches by focusing on maintaining higher-quality tracks. Data association is the key module in such architectures. In our approach, we experimented with various association criteria, from Intersection over Union (IoU) to Structural Distance IoU (SDIoU) and Generalized IoU (GIoU) with a basic linear assignment algorithm (Hungarian algorithm). We also introduced a better method to associate unconfident tracks, using our proposed fusion association technique (SAD-GWAC), which makes use of projected LiDAR depth to associate camera boxes with tracks before promoting unconfirmed tracks. Mathematical formulation and implementation details are provided for each module in the following sub-sections.

#### 3.2.1. Object Detection

Object detection is an essential task for tracking and directly impacts the tracking accuracy. A poor detector will severely hit the tracking quality, depending on the main cause of the bad performance of the detector. A high-recall detector is more likely to increase FPs even with good filtering still affecting the tracker, especially in high occlusion scenes, which can cause false breaking decisions, resulting in road accidents. A high-precision detector is more likely to misdetect objects, increasing FNs that can ignore a crossing pedestrian with low detection confidence, resulting in a direct hit. It is important to select a high-precision, high-recall detector, to reduce the required effort of handling noisy inputs. In this work, we used a common 3D detector [22] used by [9,14], as we aimed to benchmark with their work, as our tracker baseline inherits their attributes. So, to provide a fair comparison, we unified the input to our method. For the 2D detector, we used the original work of [23].

#### 3.2.2. State Estimation

A typical Kalman filter is designed for BEV-tracking, as [14] states. First, it maps the states from length, width and height 3D representation to aspect ratio and area BEV representation. This format accounts for geometry variations and is able to filter noisy detections, leading to a better stability, or gets partially occluded so it can adjust predicted states, accordingly reducing FNs and track termination that increase IDSWs. By modeling acceleration as a white noise process within the process covariance matrix, it accounts for short non-linear motion where typical assumptions of linear motion, as in standard constant velocity, will not hold, as in a sudden change in velocity or direction. Adding more intuition to that helps increase the predicted state uncertainty to include possible distant measurements.

Each tracker assigns a Kalman filter to track the box states. The Kalman filter prediction equation is the state transition matrix representing the CV motion model, with the acceleration as an additive white noise to compensate for the instantaneous change in velocity, as shown in Equation (1). The uncertainty in the tracker state represented by Equation (2) has the influence of propagating the error modeled as the change in velocity to the uncertainty in the estimated states.

(1) $${\tilde{x}}_{k}=F{\;\hat{x}}_{k-1}+v$$

(2) $${\tilde{P}}_{k}=F{\hat{P}}_{k-1}F^{T}+Q$$

Equation (3) expands each term into the matrix formula.

(3) $${\tilde{x}}_{k}=\left[\begin{array}{c} p_{x,k+1}\\ p_{y,k+1}\\ p_{\varphi ,k+1}\\ v_{x,k+1}\\ v_{y,k+1}\\ r_{k+1}\\ a_{k+1}\\ {\dot{a}}_{k+1} \end{array}\;\right]=\left[\begin{array}{cccccccc} 1 & 0 & 0 & T_{k} & 0 & 0 & 0 & 0\\ 0 & 1 & 0 & 0 & T_{k} & 0 & 0 & 0\\ 0 & 0 & 1 & 0 & 0 & 0 & 0 & 0\\ 0 & 0 & 0 & 1 & 0 & 0 & 0 & 0\\ 0 & 0 & 0 & 0 & 1 & 0 & 0 & 0\\ 0 & 0 & 0 & 0 & 0 & 1 & 0 & 0\\ 0 & 0 & 0 & 0 & 0 & 0 & 1 & 1\\ 0 & 0 & 0 & 0 & 0 & 0 & 0 & 1 \end{array}\right]\left[\begin{array}{c} p_{x,k}\\ p_{y,k}\\ p_{\varphi ,k}\\ v_{x,k}\\ v_{y,k}\\ r_{k}\\ a_{k}\\ {\dot{a}}_{k} \end{array}\right]+\left[\begin{array}{c} \frac{\Delta T^{2}}{2}w_{x,k}\\ \frac{\Delta T^{2}}{2}w_{y,k}\\ w_{\varphi ,k}\\ T_{k}w_{x,k}\\ T_{k}w_{y,k}\\ w_{r,k}\\ w_{a,\;k}\\ w_{\dot{a},k} \end{array}\right]$$

The covariance matrix is derived from Equation (4) and expanded into matrix form in Equation (5).

(4) $$Q_{k}=\mathrm{Cov}\left(w_{k}\right)=E\left[w_{k}\cdot w_{k}^{T}\right]$$

(5) $$Q_{k}=\left[\begin{array}{cccccccc} \frac{\Delta T^{4}}{4}\sigma_{w_{x}}^{2} & 0 & 0 & \frac{\Delta T^{3}}{2}\sigma_{w_{x}}^{2} & 0 & 0 & 0 & 0\\ 0 & \frac{\Delta T^{4}}{4}\sigma_{w_{y}}^{2} & 0 & 0 & \frac{\Delta T^{3}}{2}\sigma_{w_{y}}^{2} & 0 & 0 & 0\\ 0 & 0 & \sigma_{w_{\varphi }}^{2} & 0 & 0 & 0 & 0 & 0\\ \frac{\Delta T^{3}}{2}\sigma_{w_{x}}^{2} & 0 & 0 & \Delta T^{2}\sigma_{w_{x}}^{2} & 0 & 0 & 0 & 0\\ 0 & \frac{\Delta T^{3}}{2}\sigma_{w_{y}}^{2} & 0 & 0 & \Delta T^{2}\sigma_{w_{y}}^{2} & 0 & 0 & 0\\ 0 & 0 & 0 & 0 & 0 & \sigma_{w_{r}}^{2} & 0 & 0\\ 0 & 0 & 0 & 0 & 0 & 0 & \sigma_{w_{a}}^{2} & 0\\ 0 & 0 & 0 & 0 & 0 & 0 & 0 & \sigma_{\dot{a}}^{2} \end{array}\right]$$

The tracker update equations that follow the Kalman filter merge the tracked states with the new measurements. Innovation covariance is computed, representing the uncertainty in the information gain that will contribute to the states, as shown in Equation (6)

(6) $$S=H\;{\tilde{P}}_{k+1}H^{T}+R$$

The Kalman gain contributes as a weighting parameter, controlling which side to trust for each state between the new measurement and the previous state, depending on the uncertainty, as shown in Equation (7).

(7) $$K={\tilde{P}}_{k+1}H^{T}S^{-1}$$

The innovation term represents the information gain, which is the difference between the new information (measurement) and the predicted state, computed as in Equation (8).

(8) $$\gamma =(z_{k+1}-H{\tilde{x}}_{k+1})$$

Updated states and covariances based on the Kalman gain and the innovation are shown in Equations (9) and (10).

(9) $${\hat{x}}_{k+1}={\tilde{x}}_{k+1}+K\gamma$$

(10) $${\hat{P}}_{k+1}=\left(I-KH\right)\;{\tilde{P}}_{k+1}$$

The measurement model of the detection sensor is responsible for mapping the predicted states to sensor space to compute the information gain that will update the states later. It can reformat the data into a compatible form to the sensor space. Some states can be discarded, such as the states and the change in geometry, which is achieved through Equation (11) in matrix form.

(11) $$\left[\begin{array}{c} z_{p_{x},k+1}\\ z_{p_{y},k+1}\\ z_{\varphi ,k+1}\\ r\\ a \end{array}\right]=\left[\begin{array}{cccccccc} 1 & 0 & 0 & 0 & 0 & 0 & 0 & 0\\ 0 & 1 & 0 & 0 & 0 & 0 & 0 & 0\\ 0 & 0 & 1 & 0 & 0 & 0 & 0 & 0\\ 0 & 0 & 0 & 0 & 0 & 1 & 0 & 0\\ 0 & 0 & 0 & 0 & 0 & 0 & 1 & 0 \end{array}\right]\left[\begin{array}{c} p_{x,k+1}\\ p_{y,k+1}\\ p_{\varphi ,k+1}\\ v_{x,k+1}\\ v_{y,k+1}\\ r_{k+1}\\ a_{k+1}\\ {\dot{a}}_{k+1} \end{array}\right]+\left[\begin{array}{c} v_{p_{x},k+1}\\ v_{p_{y},k+1}\\ v_{\varphi ,k+1}\\ v_{r,k+1}\\ v_{a,k+1} \end{array}\right]$$

#### 3.2.3. Data Association

**Figure 3:**
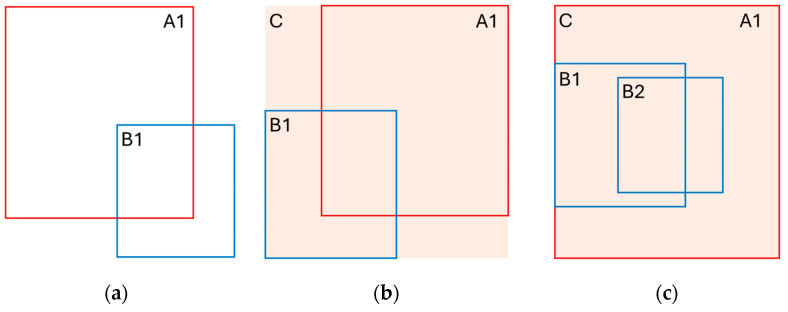
Common box association metrics. Comparing each to another to deduce the influence on the association: (**a**) typical IoU metric between box ‘A1’ and ‘B1’ that accounts for the overlapped area only; (**b**) improved modification of IoU that accounts for the minimum enclosing area ‘C’ of both boxes, ‘A1’ and ‘B1’; (**c**) special case where GIoU can be ineffective when the box to associate contains two boxes, B1 and B2, both overlapping with similar degree; in this case, it may favor ‘B1’ over ‘B2’, as it overlaps with higher value, since both boxes share the same enclosing area.

The update quality of the tracker is highly dependent on the association method between the predicted states and the new measurements. After careful review of existing box association methods, we have built a two-way association process, which will be explained in detail in the following sub-section. Figure 3 demonstrates the difference between using typical ‘IoU’ as the association metric against a generalized version of it, ‘GioU’. To build the intuition in how GioU improves association, we can think of IoU as just a metric to find the overlap between boxes, regardless of the central distance between boxes. On the other hand, GioU accounts for the enclosing area of both boxes in addition to the overlap. GioU assigns a higher matching score for boxes that have a high overlap degree and minimum enclosing area. However, in the case when one of the two boxes, ‘A1’, contains the other, ‘B1’, or even worse, when it contains two boxes, ‘B1’ and ‘B2’, each overlapping with A1 by a close value, it is hard to tell, using the IoU or the GioU, which is the best match when both IoU and GioU will select (B1) as a better match than (B2), which should not be the case if (B2) seems more fit relative to the central distance. This problem is handled by our camera fusion algorithm.

**Figure 4:**
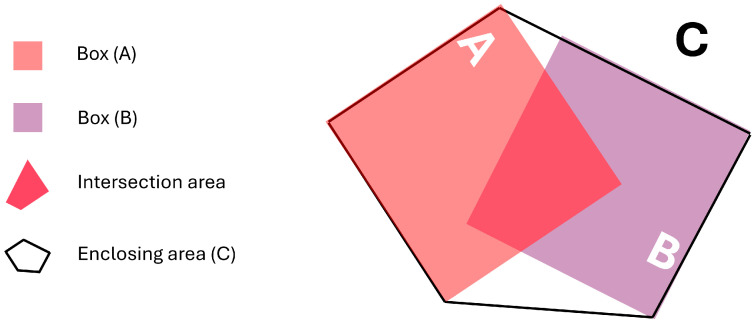
Oriented Generalized Intersection over Union (OGIoU).

LiDAR BEV-boxes are associated with tracks using oriented GioU (OGIoU), shown in Figure 4, which considers the influence of the box orientation on the intersection area in the case that it is estimated by the convex hull algorithm. For the camera case, 2D boxes are associated with the previous 2D boxes assigned to the tracks using the GioU algorithm.

### 3.3. Fusion Process

The fusion process is simply a two-step association. First, LiDAR 3D boxes associate to existing tracks and update their 3D location and size using the Kalman update equation. The second step is to associate camera 2D detection with existing tracks, confirming the existence of the tracks and updating their semantic information.

**Figure 5:**
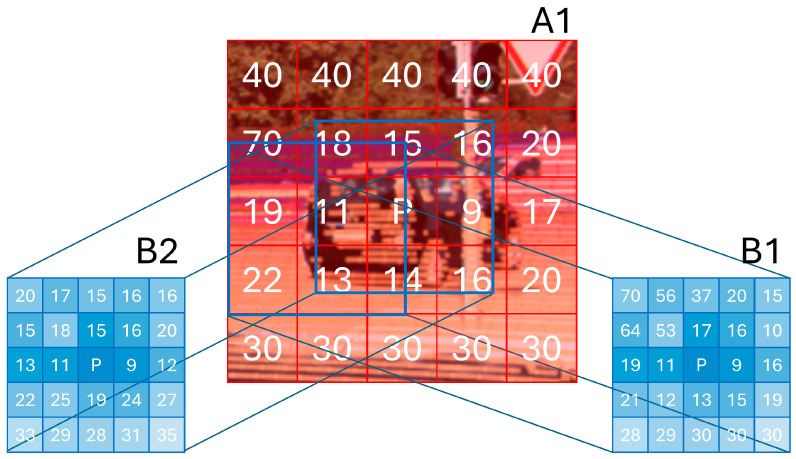
GWAC algorithm divides each box (A1, B1, B2) into grid cells. For each grid cell, the numerical value corresponding to the cell is the weighted average of all the depth values that are located inside the cell. Pixel (p) represents the box center, which is the point to compute the census transform relative to it. Then, using the Hamming distance to create the binary pattern represents the box structure.

Camera detections can increase track confidence if successfully associated with it. However, it is hard to decide which camera detections are the perfect match to the tracks, especially if the track is projected to the 2D image it can end up overlapping two camera detections each has different depth. As shown in Figure 5, the projected track is A1, which contains two camera detections (B1, B2); in this case, GIoU can lead to the wrong association. To overcome this challenge, we propose SAD-GWAC. First, we project the LiDAR point-cloud into the image. Then, we divide all the 2D boxes to be associated into grid cells and compute the corresponding depth value by calculating the weighted average among each cell, as the weighted average can handle noise and outliers and prioritizes areas of interest, putting lower weights to noise or background. Then, we compute the sum of the absolute difference between corresponding cells, and we compute the census Hamming distance of the box’s cells’ depth relative to the center pixel of the box, which acts as a structure similarity loss.

The final cost is a weighted sum between the absolute difference and the census distance. The weighting parameter controls the relative importance of the depth values and the structural pattern. This will ensure that boxes associated together are the most similar in structure and in distance to target.

The cost value of a pair of 2D boxes (track 2D projection, camera 2D detection) is calculated as in Equation (12).

(12) Cost(a, b)=α⋅ AD(a, b)+(1−α)⋅Census(a, b)

The similarity cost between box grids (a, b) is that the ‘Census’ function takes the boxes’ (a, b) grid cells where the binary value corresponds to the (N_cells − 1) per box.

### 3.4. Track Management

The track manager is responsible for managing the track’s life cycle from initialization to termination. Depending on the input measurement, it uses the corresponding association criterion: when it receives LiDAR 3D boxes, it uses OGIoU for BEV-tracks, and for the camera 2D boxes, it uses GIoU. First, it checks the two-step association to existing tracks, updating their states, and the unmatched detections (LiDAR and camera) are used to check the tentative tracks that got occluded in the previous cycles. If they are successfully matched, it recovers these tracks as unconfirmed tracks while unmatched detections check the association to unconfirmed tracks. Fused tracks from the unconfirmed tracks are promoted as new tracks, and unmatched detections that failed to associate with all the previous steps initialize new unconfirmed tracks, as shown in Algorithm 1.
**Algorithm 1** CLF-BEVSORT Track Manager1:**Data:**2:Track list *T* = {*t*_0_, *t*_1_, *…*, *t_k_*}3:Unconfident Track list *U* = {*u*_0_, *u*_1_, *…*, *u_g_*}4:Lost Track list *L* = {*l*_0_, *l*_1_, *…*, *l_h_*}5:**Input:**6:3D LiDAR detections *D*_3*d*_ = {*d*_0_, *d*_1_, *…*, *d_n_*}7:2D Camera detections *D*_2*d*_ = {*d*_0_, *d*_1_, *…*, *d_m_*}8:**Output:** *T*9:**Initialize:**10:*T* ← *φ*11:*U* ← *φ*12:*L* ← *φ*13:for frame *f* in *F* do14:   Associate *T^f^*_3*d*_, *D^f^*_3*d*_ based on GIoU(*D^f^_bev_*, *T^f^_bev_*)15:   Update *T*_3*d*_ given *matched*(*T*_3*d*_,*D*_3*d*_)16:   Associate *T^f^*_2*d*_, *D^f^*_2*d*_ based on GIoU(*D^f^*_2*d*_, *T^f^*_2*d*_)17:   Fuse *T*_3*d*_ with *D*_2*d*_ given *matched*(*T*_3*d*_,*D*_2*d*_)18:   *D^f^*_3*d*_ ← *unmatched D^f^*_3*d*_19:   *D^f^*_2*d*_ ← *unmatched D^f^*_2*d*_20:   Associate *L^f^*_3*d*_, *D^f^*_2*d*_ based on GWAC_Affinity(*D^f^*_2*d*_, *L^f^*_2*d*_)21:   Recover *L*_3*d*_ given *matched*(*L*_3*d*_, *D*_2*d*_): *recovered* ∈ *U*22:   
/* from lost track stage */
23:   *D^f^*_2*d*_ ← *unmatched D^f^*_2*d*_24:   Associate *U^f^*_3*d*_, *D^f^*_3*d*_ based on GIoU(*D^f^_bev_*, *U^f^_bev_*)25:   Update *U*_3*d*_ given *matched*(*U*_3*d*_, *D*_3*d*_)26:   Associate *U^f^*_2*d*_, *D^f^*_2*d*_ based on GIoU(*D^f^*_2*d*_, *U^f^*_2*d*_)27:   Fuse *U*_3*d*_ with *D*_2*d*_ given *matched*(*U*_3*d*_, *D*_2*d*_)28:   Upgrade Fused *U*_3*d*_ into *new tracks*: *new track* ∈ *T*29:   ∀*u*: *u* ∈ *U* delete *t* if *age* ≥ *max age*30:   ∀*l*: *l* ∈ *L* delete *t* if *age* ≥ *max age*31:**end for**32:**Return:** 
*T*


## 4. Evaluation

This section briefly mentions the dataset being used in this work, highlighting its characteristics and challenges and explaining how it can influence the results, with further detail on the benchmark evaluation metrics and their contribution to the main evaluation metric.

### 4.1. Dataset

The KITTI dataset [24] is a popular benchmark dataset used for evaluating autonomous driving algorithms with multiple sensors (cameras, 64-bit LiDAR, GPS and IMU). For the 3DMOT challenge dataset, it has 21 training sequences and 29 test sequences with more than 7400 training images and 7500 test images, with their corresponding point clouds containing more than 80K labeled objects. Despite the fact that the data have eight labeled different classes, namely, ‘Car’, ‘Pedestrian’, ‘Cyclist’, ‘Van’, ‘Truck’, ‘Tram’ and ‘Misc, the only classes used in their official benchmark are ‘Car’ and ‘Pedestrian’, as they consider these classes to have enough instances for a comprehensive evaluation. The main evaluation metric used to assess the track quality is ‘HOTA’, which is evaluated on the projection of the 3D tracks on the image as 2D tracks. One of the challenges included in this dataset is the occlusion, which is divided into three categories, ‘Fully Visible,’ ‘Partially Occluded’ and ‘Difficult’, with a defined truncation ratio for each that describes the fraction of objects lying outside the image boundary. The Fully Visible category has a maximum truncation of 15% and minimum box height of 40 pixels. The Partially Occluded category has a maximum truncation of 30% and a minimum box height of 25 pixels. The Difficult occlusion category has a maximum truncation of 50% and minimum box height of 25 pixels. It is notable that some of the ground truth annotations of the 3D box’s location are very challenging for the state-of-the-art detectors to detect due to high occlusion, which will reflect some increase in the number of false negatives that will impact the evaluation metrics.

### 4.2. Evaluation Metrics

Object Tracking Accuracy (MOTA) measures the overall accuracy of the tracking system, considering FPs, FNs and IDWS. Multiple Object Tracking Precision (MOTP) measures the precision of the predicted object locations. Multiple Object Detection Accuracy (MODA) measures the accuracy of object detection. Hits Overlaps Tracking Accuracy (HOTA) is the main metric for evaluation on the KITTI benchmark, as it provides a more comprehensive evaluation that combines the strengths of MOTA and MOTP considering the number of correctly tracked objects, the average overlap between predicted and ground truth bounding boxes and how well the tracker maintains the object identities. Clear Recall (CLR_Re) measures the proportion of ground truth objects that were correctly tracked. Clear Precision (CLR_Pr) measures the proportion of predicted objects that were correct. Missed Tracks Ratio (MTR) measures the percentage of ground truth objects that were not tracked. Partly Tracked Ratio (PTR) measures the percentage of ground truth objects that were partially tracked (i.e., tracked for some frames but not for the entire sequence). Mostly Lost Ratio (MLR) measures the percentage of ground truth objects that were lost for a significant portion of their lifespan. Smoothed MOTA (sMOTA) is a smoothed version of MOTA that accounts for the temporal distribution of errors. Clear True Positives (CLR_TP) are the number of correctly tracked objects. Clear False Negatives (CLR_FN) are the number of missed objects. Clear False Positives (CLR_FP) are the number of incorrectly predicted objects. Identity Switch (IDSW) is the number of times an existing track was incorrectly assigned to a new object. Missed Tracks (MT) are the total number of missed objects. Partly Tracked (PT) is the total number of partially tracked objects. Mostly Lost (ML) is the total number of objects lost for a significant portion of their lifespan.

## 5. Results

In this section, we provide comprehensive exploitation of the results against the recent similar work that relies on classical fusion. As shown in Table 1, our model exceeds previous, similar methods that rely on either LiDAR classical tracking methods or classical camera LiDAR fusion methods based on KITTI HOTA scores. Table 1 and Table 2 show the HOTA score and its dependent metrics for the ‘Car’ and ‘Pedestrian’ classes, respectively. The HOTA score of 77.26% for cars and 46.03% for pedestrians demonstrates the robustness of CLF-BEVSORT’s fusion strategy, particularly in challenging tracking scenarios, where single-modality approaches like AB3DMOT show significantly lower scores.

Table 3 and Table 4 exploit the effect of our fusion method against the vanilla baselines that our work extends, highlighting the contributions and limitations it brings for the ‘Car’ and ‘Pedestrian’ classes, respectively, which are discussed in detail in the following section. These metrics do not directly contribute to the HOTA score; however, they reflect how reliable and efficient the model is.

While CLF-BEVSORT does not achieve the highest precision in some cases, the balance between precision AssPr (90.35%) and recall AssRe (80.45%) ensures consistent tracking accuracy, contributing to the highest HOTA scores among evaluated models. Compared to the LiDAR-only BEVSORT framework, CLF-BEVSORT improves pedestrian HOTA by 17.91%, underscoring the value of integrating semantic information from camera data to address LiDAR sparsity.

## 6. Discussion

As shown in Table 3 and Table 4, our method achieves the lowest IDSW across both the ‘Car’ and ‘Pedestrian’ classes, reflecting its ability to maintain consistent object tracking. This improvement stems from our novel association method, which leverages 3D structural similarity to accurately match fused 2D camera detections with tracked 3D objects. By considering the structural features in the LiDAR depth map, the method effectively mitigates mis-associations, especially in challenging scenarios involving crossing objects or partial occlusions, where traditional methods often fail. The reduction in IDSW ensures more stable and reliable tracked object dynamics, a critical factor in autonomous driving scenarios.

For the ‘Car’ class, our method reduces missed tracks (MT) and partially tracked objects (PT) by approximately 19% and 36%, respectively, compared to the vanilla baseline. These improvements highlight the robustness of our approach in leveraging the distinct structural variations of cars in the LiDAR depth map. However, the results also indicate a 5% increase in most lost tracks (ML), which can be attributed to stricter association constraints in our fusion strategy. This trade-off reflects a focus on reducing false positives, leading to more reliable detections at the cost of occasionally losing objects during extended occlusions.

For the ‘Pedestrian’ class, the results show some limitations in MT, PT and ML compared to the vanilla baselines. These limitations are likely due to our association method’s tendency to alternate object states between confident and lost, especially for small, distant objects with sparse LiDAR representations. Despite these challenges, our method demonstrates reliable performance in IDSW, reinforcing its robustness in maintaining identity consistency even under occlusion-heavy scenarios. Additionally, the fusion association strategy, while effective at suppressing false positives, may inadvertently reduce the number of PT tracks and increase ML rates for smaller, less distinct pedestrian objects.

**Figure 6:**
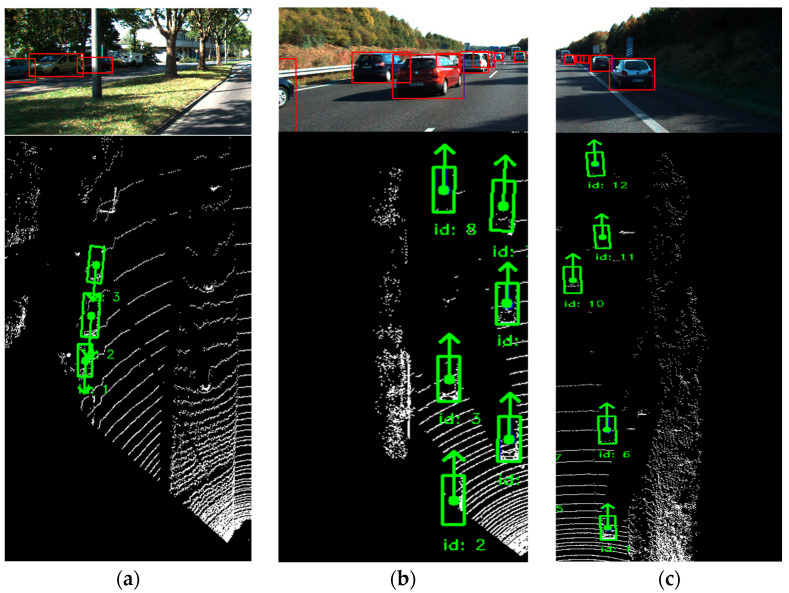
Demonstration of our reliable tracking method under partial occlusion, with arrows indicating the direction of motion of the tracked object: (**a**) tracked object with ID ‘3’ represents a simple occlusion case that typical methods can be robust against; (**b**) tracked object with ID ‘8’ represents a more challenging occlusion case, which our method was able to keep track of by successfully merging with camera detection; (**c**) represents a hard occlusion case for track ID ‘12’, highlighting the contribution of our association method for recovering a lost object undergoing a short duration hard occlusion.

Despite these minor trade-offs, our method achieves a significant improvement in HOTA, with an 18% higher score for pedestrians compared to the best vanilla baseline. This substantial gain underscores the effectiveness of our approach in balancing precision, recall and association reliability, even in the presence of challenges like occlusions and visually ambiguous objects. To showcase the effectiveness of our method in challenging dynamic scenes with occlusions, Figure 6 highlights successful tracking scenarios. These include maintaining tracks during brief occlusions, handling partially occluded objects by mitigating camera depth limitations and successfully recovering objects after short but challenging occlusions, demonstrating the robustness of our association method.

While CLF-BEVSORT delivers significant advancements in multi-object tracking (MOT) through its fusion of LiDAR and camera data in bird’s eye view (BEV) space, certain limitations remain, offering avenues for future exploration and refinement. One limitation lies in the motion model employed. To maintain computational efficiency suitable for real-time embedded systems, CLF-BEVSORT relies on a constant velocity (CV) model implemented with a linear Kalman filter. While this approach achieves high accuracy and even surpasses some deep learning-based methods, it may not fully capture complex object dynamics such as sudden accelerations or non-linear trajectories.

Higher-order models, such as the constant acceleration (CA) or constant turn rate and velocity (CTRV) models, paired with Extended Kalman Filters (EKF) or Unscented Kalman Filters (UKF), could potentially offer enhanced performance by better modeling non-linear motions. However, these approaches would introduce additional computational overhead, which might not be suitable for resource-constrained environments. Future research could investigate the trade-offs between computational cost and tracking accuracy by comparing these advanced models within similar fusion frameworks. Another potential improvement lies in the sensor fusion strategy. CLF-BEVSORT employs a fixed association cost function based on structural similarity metrics and does not dynamically adjust the contribution of each sensor modality. An adaptive fusion mechanism, where the weight of LiDAR and camera data dynamically adjusts based on environmental conditions (e.g., lighting, weather or object distance), could further enhance robustness. For instance, in low-light scenarios, LiDAR data might be prioritized, while camera data could dominate in high-visibility conditions.

Incorporating deep learning models for feature extraction represents another promising direction. Instead of relying solely on structural similarity for association, learned feature embeddings could enable the system to differentiate between visually similar objects more effectively. Importantly, this approach does not necessitate end-to-end deep learning models for tracking, preserving the modularity of the system while leveraging the power of deep learning for specific tasks like object appearance modeling or similarity scoring. Additionally, the current method relies on projecting 3D LiDAR data into 2D image space to include depth information in the structural descriptor for association. An alternative approach could leverage a stereo camera setup to infer depth directly from camera data, eliminating the need for projection. This inferred depth could then be integrated into the BEV space and used in the association process. Such a method might enhance spatial accuracy, reduce sensitivity to occlusions, and further minimize identity switches (IDSWs).

Lastly, future work could explore how the SAD-GWAC (Sum Absolute Difference Grid Weighted Average Census) metric performs under different configurations or with additional descriptors. Variations in grid granularity, feature weighting or hybrid similarity metrics could potentially enhance tracking accuracy in complex, cluttered environments. In summary, while CLF-BEVSORT effectively balances accuracy and efficiency, future research could focus on more sophisticated motion models, adaptive fusion strategies, integration of deep learning features and alternative methods for depth inference. The system can be tested with other dataset that include harsh weather conditions where the sensors can have limited visibility. These directions have the potential to further improve robustness, scalability and real-world applicability for autonomous vehicle tracking systems.

## 7. Conclusions

CLF-BEVSORT presents a robust and efficient framework for multi-object tracking in self-driving cars, effectively harnessing the complementary strengths of camera and LiDAR sensors. By incorporating structural similarity between camera and LiDAR detections, the proposed association method significantly enhances tracking accuracy, particularly in challenging scenarios with occlusions and dynamic environments. CLF-BEVSORT ensures stable and reliable object tracking by minimizing ID switches and reducing missed tracks, thereby contributing to the overall safety and performance of autonomous vehicles. The method’s demonstrated effectiveness in handling various occlusion cases underscores its potential for real-world applications, making it a valuable asset to the field of self-driving car perception. The proposed framework offers several advantages. Effective fusion of camera and LiDAR data provides resilience to sensor limitations and environmental challenges, ensuring robustness. Incorporating structural similarity in the association step leads to more reliable tracking results, enhancing accuracy. The SORT tracking framework, combined with the proposed association method, ensures efficient and simple implementation, contributing to overall efficiency. In conclusion, CLF-BEVSORT represents a new, efficient way to fuse camera and LiDAR detections, considering both semantics for multi-object tracking for self-driving cars, offering a robust, accurate and efficient solution that can enhance the safety and performance of autonomous vehicles.

## Figures and Tables

**Table 1:** KITTI tracking benchmark for the ‘Car’ class on HOTA metrics.

Model	HOTA ↑	DetA ↑	AssA ↑	DetRe ↑	DetPr ↑	AssRe ↑	AssPr ↑
AB3DMOT	69.99%	71.13%	69.33%	75.66%	84.40%	72.31%	89.02%
BEVSORT	71.66%	66.95%	76.91%	75.11%	80.45%	80.30%	89.73%
CenterTrack	73.02%	**75.62%**	71.20%	**80.10%**	84.56%	73.84%	89.00%
EagerMOT	74.39%	75.27%	74.16%	78.77%	**86.42%**	76.24%	**91.05%**
DeepFusionMOT	75.46%	71.54%	**80.05%**	75.34%	85.25%	**82.63%**	89.77%
Mono 3D KF	75.47%	74.10%	77.63%	78.86%	82.98%	80.23%	88.88%
StrongFusionMOT	75.65%	72.08%	79.84%	75.20%	86.23%	82.42%	89.81%
**CLF-BEVSORT**	**77.26%**	74.76%	79.88%	75.57%	85.13%	80.45%	90.35%

↑ indicates that higher values correspond to better performance, whereas ↓ indicates that lower values correspond to better performance. This format applies to all the tables.

**Table 2:** KITTI tracking benchmark for the ‘Pedestrian’ class on HOTA metrics.

Model	HOTA ↑	DetA ↑	AssA ↑	DetRe ↑	DetPr ↑	AssRe ↑	AssPr ↑
AB3DMOT	37.81%	32.37%	44.33%	34.91%	59.35%	48.44%	62.83%
BEVSORT	39.04%	32.71%	46.81%	43.95%	45.15%	49.71%	68.03%
CenterTrack	40.35%	**44.48%**	36.93%	**49.91%**	66.83%	41.05%	**70.19%**
EagerMOT	39.38%	40.60%	38.72%	43.43%	61.49%	40.98%	68.33%
Be-Track	43.36%	39.99%	47.23%	43.00%	69.03%	51.28%	69.60%
Mono 3D KF	42.87%	40.13%	46.31%	46.02%	59.91%	**52.86%**	63.50%
StrongFusionMOT	43.42%	38.86%	48.83%	45.30%	53.81%	52.54%	63.17%
**CLF-BEVSORT**	**46.03%**	42.76%	**51.88%**	48.57%	**69.13%**	51.45%	69.35%

Bold value means it is the best value for the corresponding metric among all models. This format applies to all the tables.

**Table 3:** Comprehensive evaluation incorporating missing and hitting rates for the ‘Car’ class.

Model	IDSW ↓	MT ↓	PT ↑	ML ↓	MTR ↓	PTR ↑	MLR ↓
AB3DMOT	62	304	147	113	53.90	26.06	20.03
BEVSORT	57	316	185	**63**	56.02	32.80	**11.17**
**CLF-BEVSORT**	**31**	**246**	**252**	66	**43.61**	**44.68**	11.70

**Table 4:** Comprehensive evaluation incorporating missing and hitting rates for the ‘Pedestrian’ class.

Model	IDSW ↓	MT ↓	PT ↑	ML ↓	MTR ↓	PTR ↑	MLR ↓
AB3DMOT	145	**42**	66	59	**25.15**	39.52	35.33
BEVSORT	148	44	**71**	**52**	26.34	**42.51**	**31.13**
**CLF-BEVSORT**	**89**	73	36	58	43.71	31.55	34.73

## Data Availability

Data available in a publicly accessible repository.

## Abbreviations

The following abbreviations are used in this manuscript:

IoU	Intersection over Union
OGIoU	Oriented Generalized Intersection over Union
KF	Kalman Filter
CV	Constant Velocity
ToF	Time of Flight
MOT	Multi Object Tracking
ADAS	Advanced Driving Assistance Systems
BEV	Bird’s Eye View
TTC	Time to Collision
IDSW	Identity Switching
FP	False Positive
FN	False Negative

## References

[1] Bewley A., Ge Z., Ott L., Ramos F., Upcroft B. Simple Online and Realtime Tracking. Proceedings of the International Conference on Image Processing, ICIP 2016.

[2] Zhou X., Koltun V., Krähenbühl P. Tracking Objects as Points. Proceedings of the Computer Vision—ECCV 2020: 16th European Conference.

[3] Cao J., Pang J., Weng X., Khirodkar R., Kitani K. Observation-Centric SORT: Rethinking SORT for Robust Multi-Object Tracking. Proceedings of the 2023 IEEE/CVF Conference on Computer Vision and Pattern Recognition (CVPR).

[4] Zhang Y., Sun P., Jiang Y., Yu D., Weng F., Yuan Z., Luo P., Liu W., Wang X. (2021). ByteTrack: Multi-Object Tracking by Associating Every Detection Box. arXiv.

[5] Wojke N., Bewley A., Paulus D. Simple Online and Realtime Tracking with a Deep Association Metric. Proceedings of the International Conference on Image Processing, ICIP 2018.

[6] Shi K., Shi Z., Yang C., He S., Chen J., Chen A. (2022). Road-Map Aided GM-PHD Filter for Multivehicle Tracking with Automotive Radar. IEEE Trans. Ind. Inform..

[7] Kamann A., Steinhauser D., Gruson F., Brandmeier T., Schwarz U.T. (2021). Extended Object Tracking Using Spatially Resolved Micro-Doppler Signatures. IEEE Trans. Intell. Veh..

[8] Braun M., Luszek M., Meuter M., Spata D., Kollek K., Kummert A. Deep Learning Method for Cell-Wise Object Tracking, Velocity Estimation and Projection of Sensor Data over Time. Proceedings of the International Conference on Intelligent Transportation Systems (ITSC).

[9] Weng X., Wang J., Held D., Kitani K. (2020). AB3DMOT: A Baseline for 3D Multi-Object Tracking and New Evaluation Metrics. arXiv.

[10] Wu H., Han W., Wen C., Li X., Wang C. (2022). 3D Multi-Object Tracking in Point Clouds Based on Prediction Confidence-Guided Data Association. IEEE Trans. Intell. Transp. Syst..

[11] Choi J., Ulbrich S., Lichte B., Maurer M. Multi-Target Tracking Using a 3D-Lidar Sensor for Autonomous Vehicles. Proceedings of the IEEE Conference on Intelligent Transportation Systems, Proceedings (ITSC 2013).

[12] Pang Z., Li Z., Wang N. (2021). SimpleTrack: Understanding and Rethinking 3D Multi-Object Tracking.

[13] Liu J., Bai L., Xia Y., Huang T., Zhu B., Han Q.-L. (2023). GNN-PMB: A Simple but Effective Online 3D Multi-Object Tracker without Bells and Whistles. IEEE Trans. Intell. Veh..

[14] Alfeqy L., Abd El Munim H.E., Maged S.A., Mohamed D. BEVSORT: Bird Eye View LiDAR Multi Object Tracking. Proceedings of the 2024 IEEE 22nd Mediterranean Electrotechnical Conference, MELECON 2024.

[15] Wang X., Fu C., He J., Wang S., Wang J. (2023). StrongFusionMOT: A Multi-Object Tracking Method Based on LiDAR-Camera Fusion. IEEE Sens. J..

[16] Wang X., Fu C., Li Z., Lai Y., He J. (2022). Deep FusionMOT: A 3D Multi-Object Tracking Framework Based on Camera-LiDAR Fusion With Deep Association. IEEE Robot. Autom. Lett..

[17] Kim A., Osep A., Leal-Taixé L. Eagermot: 3D Multi-Object Tracking via Sensor Fusion. Proceedings of the 2021 IEEE International Conference on Robotics and Automation (ICRA).

[18] Dimitrievski M., Veelaert P., Philips W. (2019). Behavioral Pedestrian Tracking Using a Camera and LiDAR Sensors on a Moving Vehicle. Sensors.

[19] Liu Z., Tang H., Amini A., Yang X., Mao H., Rus D.L., Han S. BEVFusion: Multi-Task Multi-Sensor Fusion with Unified Bird’s-Eye View Representation. Proceedings of the 2023 IEEE International Conference on Robotics and Automation (ICRA).

[20] Munkres J. (1957). Algorithms for the Assignment and Transportation Problems. J. Soc. Ind. Appl. Math..

[21] Mei X., Sun X., Zhou M., Jiao S., Wang H., Zhang X. On Building an Accurate Stereo Matching System on Graphics Hardware. Proceedings of the IEEE International Conference on Computer Vision Workshops (ICCV).

[22] Shi S., Wang X., Li H. PointRCNN: 3D Object Proposal Generation and Detection from Point Cloud. Proceedings of the IEEE Conference on Computer Vision and Pattern Recognition (CVPR).

[23] Varghese R., Sambath M. YOLOv8: A Novel Object Detection Algorithm with Enhanced Performance and Robustness. Proceedings of the 2024 International Conference on Advances in Data Engineering and Intelligent Computing Systems.

[24] Geiger A., Lenz P., Urtasun R. Are We Ready for Autonomous Driving? The KITTI Vision Benchmark Suite. Proceedings of the 2012 IEEE Conference on Computer Vision and Pattern Recognition (CVPR).

